# Supplementary material for: Cutaneous Nevoid Melanoma: A Retrospective Study on Clinico-Pathological Characteristics, with a Focus on Dermoscopic Features and Survival Analysis
Source: Cancers (Basel). 2024 Dec 29;17(1):65. doi: 10.3390/cancers17010065 (PMC11719877; doi:10.3390/cancers17010065)
Supplement: Supplementary file 1 [file cancers-17-00065-s001.zip › cancers-3352784-supplementary.pdf]

Table S1. Follow up data, n=100.

	Overall (N=100)
**Local skin recurrence**	
local recurrence	2
N-Miss	98
**Regional lymph node recurrence**	
regional lymph node	2
N-Miss	98
**Regional skin/intransit recurrence**	
regional skin/intransit	7
N-Miss	93
**Local, in transit or regional lymph node recurrences, cumulative n. patients**	
regional skin/intransit	9
**Distant metastasis**	
distant metastatic	11
N-Miss	89
**Follow up - days**	
Median	1917.50
Range	0.00 - 7311.00
Mean (SD)	2042.89 (1757.80)
**Last vital status**	
alive	84 (84.8%)
deceased	14 (14.1%)
N-Miss	2 (2.0%)
**Cause of death**	
melanoma	10 (83.3%)
other	2 (8.4%)
N-miss	2 (8.4%)

Table S2. ABCDE features.

**N°**	**A (asimmetry)**	**B (Irregular bord)**	**C (color)**	**D (diameter)**	**E (evolving)**
1	*no*	*no*	*no*	*yes*	*yes*
2	*yes*	*yes*	*yes*	*yes*	*yes*
3	*yes*	*yes*	*yes*	*yes*	*yes*
4	*no*	*no*	*no*	*no*	*yes*
5	*yes*	*yes*	*yes*	*yes*	*yes*
6	*yes*	*yes*	*no*	*no*	*no*
7	*no*	*no*	*no*	*no*	*yes*
8	*no*	*no*	*no*	*no*	*yes*
9	*no*	*no*	*no*	*no*	*yes*
10	*no*	*no*	*no*	*yes*	*no*
11	*no*	*no*	*no*	*yes*	*yes*
12	*yes*	*yes*	*yes*	*yes*	*yes*
13	*no*	*no*	*no*	*no*	*yes*
14	*no*	*no*	*no*	*yes*	*yes*
15	*yes*	*yes*	*yes*	*yes*	*yes*
16	*no*	*no*	*yes*	*yes*	*yes*
17	*yes*	*no*	*yes*	*yes*	*yes*
18	*no*	*no*	*no*	*no*	*yes*
19	*no*	*no*	*no*	*no*	*yes*
20	*no*	*no*	*yes*	*yes*	*yes*
21	*yes*	*yes*	*yes*	*yes*	*yes*
22	*yes*	*yes*	*yes*	*yes*	*yes*
23	*yes*	*yes*	*yes*	*yes*	*yes*
24	*no*	*no*	*no*	*no*	*yes*
